# Drain-Site Hernia Containing the Vermiform Appendix: Report of a Case

**DOI:** 10.1155/2013/198783

**Published:** 2013-06-04

**Authors:** Markus Gass, Anatol Zynamon, Markus von Flüe, Ralph Peterli

**Affiliations:** ^1^Department of Surgery, St. Claraspital Basel, 4058 Basel, Switzerland; ^2^Department of Radiology, St. Claraspital Basel, 4058 Basel, Switzerland

## Abstract

The herniated vermiform appendix has been described as content of every hernia orifice in the right lower quadrant. While the femoral and inguinal herniated vermiform appendix is frequent enough to result in an own designation, port-site or even drain-site hernias are less frequently described. We report the case of a 62-year-old woman who presented with right lower quadrant pain seven years after Roux-en-Y Cystojejunostomy for a pancreatic cyst. CT scan showed herniation of the vermiform appendix through a former drain-site. A diagnostic laparoscopy with appendectomy and direct closure of the abdominal wall defect combined with mesh reinforcement was performed. Despite the decreasing use of intraperitoneal drains over the recent years, a multitude of patients had intraperitoneal drainage in former times. These patients face nowadays the risk of drain-site hernias with sometimes even unexpected structures inside.

## 1. Introduction

Herniation of the vermiform appendix has been topic of several case reports and case series [1–3]. It has been described as content of any kind of right lower quadrant abdominal wall defect, for example, inguinal, femoral, Spigelian, or incisional hernia. 

In contrast to these publications, descriptions of port- or drain-site hernias containing the vermiform appendix are scarce [4].

The usage of intraperitoneal drains has become less and less popular over the last years. Arguments for omittance of drains are a variety of postdrainage complications; some authors even claim that drains are more disadvantageous than they are beneficial [5]. Possible complications are ascending infections along the tube or arrosions of intraabdominal organs, such as the small intestine or the colon. Moreover, even in the absence of complications, patients with intraperitoneal drainage failed to have outcomes superior to those without drains [6, 7]. A complication not to be underestimated is hernia formation after removal of the drain with early evisceration or later herniation of intestinal structures [8, 9]. Obviously, the risk for those complications correlates with the drain diameter which reflects the abdominal wall defect. We describe the rare finding of a herniated vermiform appendix through a drain-site years after the initial operation.

## 2. Case Report

A 62-year-old woman underwent open Roux-en-Y Cystojejunostomy for pancreatic pseudocyst with insertion of a drain through the right lower abdominal quadrant. Seven years later, she presented with a three-week history of abdominal cramps, intermittent fever episodes, and nausea with vomiting. Antibiotic therapy was initiated, relieving the symptoms, but unspecific burning sensations in the right iliac fossa persisted. Clinical examination showed an obese patient with tenderness and pain in the right lower abdominal quadrant with localized peritonitis.

**Figure 1:**
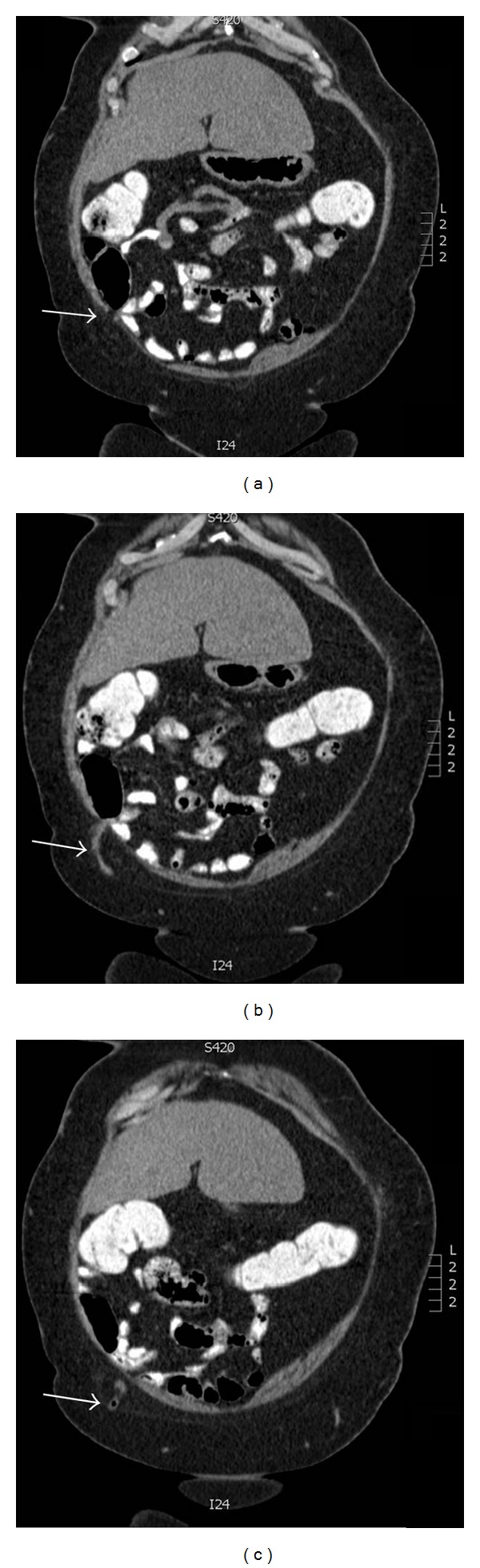
CT findings of the subcutaneous vermiform appendix. (a) Arrow indicating hernia orifice. (b) Arrow indicating subcutaneous position of vermiform appendix. (c) Arrow indicating a bubble of air in the tip of the vermiform appendix.

**Figure 2:**
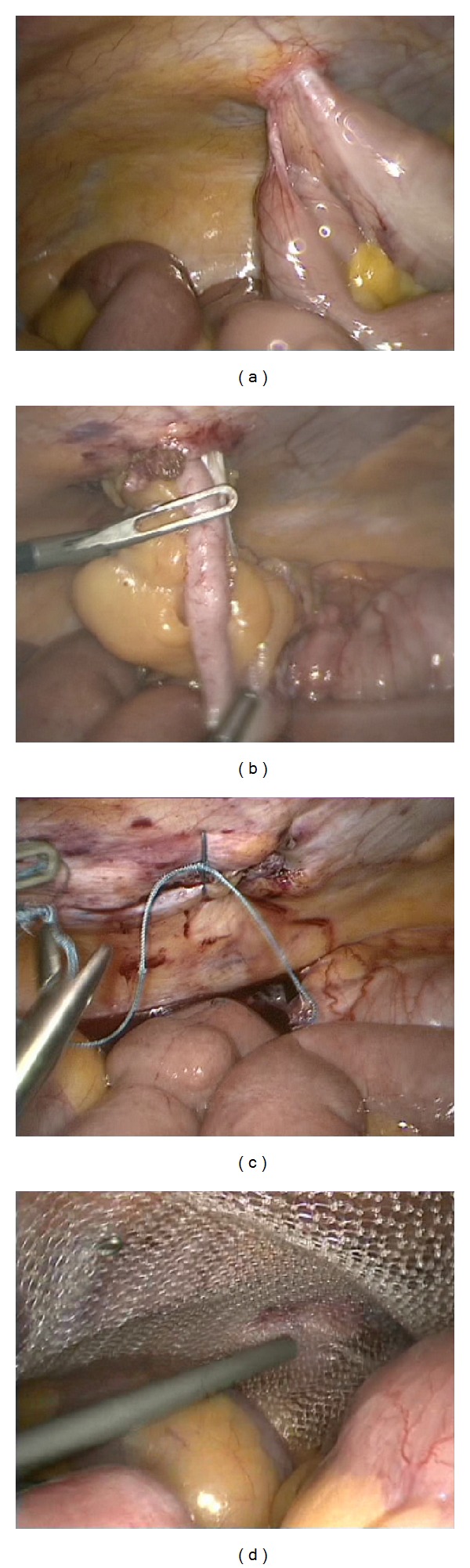
Laparoscopic views. (a) Vermiform appendix attached to the hernia orifice. (b) Reposition of vermiform appendix. (c) Defect closure with suture. (d) Augmentation with intraperitoneal onlay mesh.

A CT scan revealed a subcutaneous vermiform appendix with signs of acute inflammation (Figure 1). Laparoscopy confirmed the diagnosis: the vermiform appendix and the cecal pole up to Bauhin's valve were ensnared in a hernia orifice measuring 3 cm in diameter. The hernia content was freed, a stapler appendectomy performed and, after laparoscopic closure of the fascial defect with interrupted sutures, the defect was reinforced with a 15 cm × 15 cm Dynamesh IPOM fixed with absorbable tacks (Figure 2). The patient stayed in the hospital for seven days, mainly for her comorbidities and was discharged after an uneventful course.

## 3. Discussion

Several drain-related complications have been reported over the past years. Drain-site hernia containing the vermiform appendix is a very rare complication; only three case reports could be retrieved in a Medline search, one of them describing an evisceration. 

Duraker and colleagues reported an evisceration of the vermiform appendix which was attached to a sidehole of a drainage [4]. The stab wound was extended and appendectomy under general anesthesia performed. Even incarcerated small bowel has been described [10] or an evisceration of the gallbladder [11]. Our patient presented seven years after initial surgery with a drain-site hernia containing the vermiform appendix. It must be assumed that this hernia had been asymptomatic for a long time. Interestingly, histological examination failed to show any inflammatory signs. 

Nowadays, the use of routine drainage has become more and more unpopular since advantages are questionable and a multitude of mainly infectious complications have been described. Nevertheless, many patients after operations during the last decades suffer from drain-site scars and hernias; therefore, this case report draws attention to a common problem. We suggest reducing the usage of intraperitoneal drainage to an absolute minimum to avoid such complications. Routine use in, for example, laparoscopic gynaecological operations or laparoscopic cholecystectomies, should be omitted [12]. If needed, for example, in hepatobiliary surgery, the insertion of drains should be performed transverse through the abdominal wall to achieve a coulisse effect when pulled out. In the era of laparoscopic colorectal surgery we suggest diverting the drain, preferably through a port site no larger than 5 mm. If, for specific reasons, the drain is diverted through any larger port site, a single narrowing fascial suture or even a purse-string suture is recommended. Furthermore, small-calibre drains should be used to reduce the abdominal wall defect to a minimum. 

Similar to the mechanism of drain-site hernias, port-site hernias are described more and more frequently in the literature. Although they usually contain omental fat or small bowel, three cases of the vermiform appendix being present in a symptomatic port-site hernia have been reported [1, 13, 14]. Port-site hernias in general are reported to occur with an incidence of 0.74%, depending on the type of laparoscopic surgery performed. Independent risk factors are obesity, surgical site infection, and cutting blade trocars compared to conical tip-shaped ones [15]. The size of the inserted ports plays a pivotal role as well: the larger the diameter, the more frequently occurrence of hernias can be observed. 

We recommend laparoscopic appendectomy and hernia repair with intraperitoneal onlay mesh reinforcement for all cases of port-site hernias, drain-site hernias, or inguinal and femoral hernias containing the vermiform appendix. Laparoscopy serves as additional diagnostic tool and affords one-step minimal invasive appendectomy and hernia repair, either for drain- or port-site hernias with an onlay mesh approach, or for the inguinal or femoral hernias with the transabdominal preperitoneal inguinal hernia repair (TAPP) technique.

In conclusion routine intraperitoneal drainage should be reduced to a minimum, and potential complications must be taken into account. If definitely needed, small-calibre drains should be inserted whenever possible obliquely through the abdominal wall. 

Any abdominal wall hernia in the right lower quadrant can unexpectedly contain the vermiform appendix. If detected preoperatively a laparoscopic approach with appendectomy and hernia closure combined with intraperitoneal onlay mesh reinforcement seems to be a safe and effective approach.
